# Dental school tracks related to the retention of dentists in Thai government service: a cross-sectional survey

**DOI:** 10.1186/s12960-020-0444-7

**Published:** 2020-01-28

**Authors:** Philaiporn Vivatbutsiri, Thanachok Iempook, Sakda Wonghinkong, Sunisa Sopa, Palinee Detsomboonrat

**Affiliations:** 1 Department of Anatomy, Faculty of Dentistry, Chulalongkorn University, Bangkok, Thailand; 2 Undergraduate dental student, Bangkok, Thailand; 3 Department of Community Dentistry, Faculty of Dentistry, Chulalongkorn University, 34 Henry Dunant Road, Patumwan, Bangkok, 10330 Thailand

**Keywords:** Dental school, Admission track, Rural retention, Influencing factor

## Abstract

**Background:**

The shortage of dentists working in rural hospitals is an important public health problem resulting from dentist distribution inequity. The Ministry of Public Health of Thailand (MoPH) has implemented a policy of recruiting students with a rural background to be dental students and return home after graduating. This study aims to examine the relationship between admission tracks during the academic years 2005–2011 on retaining dentists in Thai government service and identify the factors associated with retention and resignation.

**Methods:**

A cross-sectional survey was conducted using an online questionnaire from 287 dentists who graduated from Chulalongkorn University (CU) between 2010 and 2016. Follow-up data consisted of the admission track, number of years spent in Thai government service, and factors that influenced their decision to stay or resign from Thai government service. Chi-squared analysis was used to analyze the data.

**Results:**

The overall retention rate in Thai government service was 58.2%. Dentists in the rural track had a significantly higher retention rate than the normal track (*p* = 0.023). Female dentists who were married and graduated less than 3 years had a significantly higher resignation rate than others (*p* < 0.05). The main reasons for retention were “security in the profession,” “high chance to pursue specialty training in the future,” and “close proximity to hometown.” Dentists from the CU rural admission tracks chose “close proximity to hometown” as the top reason, while others selected “security in the profession.” The main reasons influencing resignation were “workplace far away from hometown” and “getting specialty training.”

**Conclusions:**

These results indicate that dentists in the rural track had a significantly higher retention rate than the normal track. The most important factor influencing both retention and resignation was workplace location, where being near to their hometown improved the retention rate of rural dentists. Therefore, the MoPH should increase student admission into the rural track to resolve the inequity in dentist distribution.

## Background

Oral health is related to general health, both physical and mental health. Dental problems may generate pain that interferes with daily life and quality of life [1–3]. Therefore, dentists, who are doctors specializing in oral health, play an important role in the health system. However, there is a shortage in the number of oral health personnel in Thai government service, with a report in 2015 revealing that there were 13 215 dentists in Thailand, composed of 5140 dentists working in the Ministry of Public Health (MoPH), 1553 in other government services, and 6522 in the private sector. The proportion of dentists to the population in each part of Thailand was 1:1005 in Bangkok, 1:6445 in the central region, 1:6668 in the north region, 1:7181 in the south region, and 1:10 745 in the northeast region, whereas the proportion of dentists to the population nationwide was 1:4913, which demonstrated an important public health problem in the distribution of dentists, especially in rural areas [4]. Thus, the MoPH has implemented several policies over the past few decades to increase the distribution of dentists in rural areas, such as recruiting students from rural areas and sending them back to their hometown after graduation or locating dental schools outside major cities for rural students [5–7]. A policy to increase the production and distribution of graduated dentists to rural areas was introduced in 2005, and all dental schools in Thailand carried out this project from 2005 by increasing the number of dental students in the following 10 years [5].

Chulalongkorn University (CU) was the first public dental school in Thailand and implemented a special admissions policy to increase the number of admitted rural students. There are six admission tracks for CU Dental School (CUDS). These are the (i) Central University Admissions System (CUAS), which recruits dental students using a nationwide entrance examination; (ii) Direct admission for the Consortium of Thai Medical and Dental school (DCTMD), which recruits dental students directly from the Consortium of Thai Medical Schools (COMES) that aims to support and promote medical and dental health provision; (iii) the Collaborative Project to Increase Production of Rural Dentists program (CPIPRD), which recruits dental students from rural backgrounds according to the Office of Higher Education Commission (OHEC); (iv) CU Rural Admission (CURA), which aims to increase the opportunity for underprivileged students to study at CU; (v) Direct Admission for CUDS (DACD), which recruits dental students directly by CUDS; and (vi) Admission for Students in the Southern Border Provinces (ASSP) by the Ministry of Interior, which aims to increase the opportunity for students who are Thai Muslims in the Southern Border Provinces to study in any of the nine universities, including Chulalongkorn University, participating in the project of the Ministry of Interior [8].

CURA and CPIRD were included in the rural track. The aim of the CURA track was to increase the opportunity for education in rural areas and helping students in rural areas to understand the importance of education. Students recruited into the CURA track had to meet the CU criteria, including annual family income less than 120 000 baht and living in the east and the south region selected by the CURA program. The aim of the CPIPRD track was to recruit dental students from rural backgrounds who meet the criteria of the Office of Higher Education Commission. In the academic years 2005–2011, CUDS admitted approximately 140 dental students per academic year from these six admission tracks, resulting in a 2:5 ratio of dental students in the rural track and normal track. Although the rural track had special recruitment criteria, there were no differences in the education curriculum between tracks.

All dentists that graduated from public universities are assigned to work in the public sector in rural hospitals or other government services for the first 3 years after graduation. Moreover, dentists graduating from the CURA track were assigned to work in their hometown. Thus, these admission tracks had a similar purpose, to respond to the MoPH’s policy for increasing the production and distribution of dentists throughout the country [9]. Moreover, CPIPRD recruits students from a rural background to increase the distribution of dentists in rural areas through contract bonding [5, 10].

The aim of this study was to identify the associations between the admission tracks for CUDS and the subsequent retention of graduated dentists in Thai government service, which would benefit future dental manpower planning in rural areas.

## Materials and methods

This cross-sectional study was approved by the institutional review board of the Faculty of Dentistry and Human Research Ethics Committee of the Faculty of Dentistry, CU (25610418-002), and informed consent was obtained from all participants in the study. The participants’ responses were confidential and were not linked to their identity. The data were analyzed in block form, rather than individually, to ensure anonymity and confidentiality. The recruitment period started in July 2018 and ended in December 2018.

## Population and sample

This cross-sectional study was conducted using dentists who were admitted to CUDS between 2005 and 2011 (graduated 2010–2016). The minimum sample size calculation was based on a previous study [11], which reported a 54% retention of dentists in Thai government service. For estimating the finite population proportion, the number of dentists who graduated in the academic years 2005–2011 was 900 dentists and the acceptable error was 5%. Based on this, the required sample size was 269 dentists. The sample was taken from a group of dentists by consecutive sampling that relied on the data collected from dentists who were available to participate in the study until the required sample size was achieved.

## Research instruments

An online questionnaire was used to collect the data containing four parts as described below.

- Part I: Demographic characteristics of the dentist—age, sex, education level, hometown area, admission tracks, and graduation year
- Part II: Type of workplace and duration of work—government service (Thai Ministry of Public Health or Ministry of Education) or private sector
- Part III: Factors of the dentists who work in Thai government service—workplace environment, duration of work, financial factors, higher education, and family factors
- Part IV: Factors of the dentists who resigned from Thai government service—workplace environment, duration of work, financial factors, higher education, and family factors

The content validity of the questionnaire was evaluated by consulting a questionnaire expert, and a pilot test was conducted on dentists who did not meet the study’s inclusion criteria.

## Data collection

The online questionnaire was sent to the representatives of each year’s graduates, who distributed it to them, and the data were collected over 6 months. The dentists were asked about their demographic characteristics, type of workplace, and duration of working. In the last part, the dentists were asked to choose the top three factors influencing their retention or resignation (as applicable) from Thai government service. These factors were satisfaction with income, security in the profession, high chance to pursue specialty training in the future, close proximity to their hometown, and satisfactory relationships with colleagues. For each respondent, the factors that were chosen as the first, second, and third most important reasons were scored 3, 2, and 1 points, respectively. These factors were then ranked using the cumulative point score summed from all the respondents.

## Statistical analysis

The data were analyzed using the SPSS Version 22.0 software (IBM Corp.). The percentage retention of dentists in the Thai government service for each undergraduate admission tract was analyzed with descriptive statistics. The chi-squared test was used to compare influencing factors between dentists who were still working in or had resigned from Thai government service. The difference in the top three factors between dentists in the two grouping tracks was also assessed using the chi-square test.

## Results

The proportion of the 287 surveyed dentists who still worked in Thai government service was 58.2%, while 41.8% had resigned. This study examined dentists within 7 years post-graduation, 233 (81.2%) were dentists within the contracted 3-year period and 54 (18.8%) were dentists in Thai government service for more than 3 years. The average duration of work in the Thai government service was 2 years 5 months. Based on the admission track, the retention rate in CURA (88.9%) and CPIPRD (68.0%) were higher compared with the other tracks; however, this was not significant, indicating that the admission track had no significant effect on retention rate (Table 1). When considered as the two main tracks (rural and normal track), dentists in the rural track had a significantly higher retention rate compared with the normal track (*p* = 0.023). However, the retention rate of dentists in the rural track and normal track within the first 3 years in public service and after 3 years of public work was not significantly different (Table 2).

**Table 1:** Proportion (%) of recent graduate dentists who are still working or who resigned from Thai government service with respect to their admission track during the academic years 2005–2011

Admission track	Dentists working in Thai government service (%)	Dentists who resigned from Thai government service (%)	*p* value	OR (95% CI)
DCTMD	59 (56.2%)	46 (43.8%)	0.063	1
CPIPRD	34 (68.0%)	16 (32.0%)		0.604 (0.816, 3.364)
CURA	8 (88.9%)	1 (11.1%)		0.160 (0.753, 51.668)
CUAS	62 (55.9%)	49 (44.1%)		1.013 (0.576, 1.689)
DACD	4 (33.3%)	8 (66.7%)		2.564 (0.111, 1.375)
ASSP	0	0		0

*OR* odds ratio

**Table 2:** Proportion (%) of the retention rate of dentists in the rural track and normal track within the first 3-year work in public services and after 3-year public work

Track group	1–3 years			> 3 years			*p* value^c^
	Retention	Resignation	*p* value	Retention	Resignation	*p* value	
Rural track^a^	36 (72%)	14 (28%)	0.108	6 (66.7%)	3 (33.3%)	0.083	0.023
Normal track^b^	109 (59.6%)	74 (40.4%)		16 (35.6%)	29 (64.4%)		

^a^Rural track included CURA and CPIRD

^b^Normal track included DCTMD, CUAS, and DACD

^c^Significant level of all dentists in rural track and normal track

The major characteristics influencing the retention and resignation from the Thai government service are presented in Table 3, where the significant factors were sex, marital status, time since graduation, and income (*p* < 0.05). Age and having of children were not significant (*p* > 0.05). These variables (*p* < 0.25) from the bivariate analysis in the preliminary analyses were selected for the multiple logistic regression. Table 4 presents the adjusted odds ratios (ORs) and 95% confidence intervals (CIs) for the adjusted ORs of the final model for resignation from Thai government service. Sex, marital status, time since graduation, and income still significantly affected resignation after adjusting for other variables. Being in the female and marriage groups had an OR of 3.39 and 37.19, respectively, signifying that the chance of resignation would be 3.39- and 37.19-fold higher than that of the male and not marriage group, respectively. Moreover, dentists who graduated within 3 years had a 16.52-fold higher tendency to resign from Thai government service compared with dentists who graduated more than 3 years earlier.

**Table 3:** General characteristics of recent dental graduates from CUSD influencing their retention or resignation from Thai government service

Factors	Number of dentists who work in Thai government service (%)	Number of dentists who resigned from Thai government service (%)	*p* value	OR (95% CI)
Sex				
Male	59 (67.8%)	28 (32.2%)	0.029	1
Female	108 (54.0%)	92 (46.0%)		1.790 (1.058, 3.046)
Age				
20–25 years	18 (60.0%)	12 (40.0%)	0.426	1
26–30 years	137 (59.3%)	94 (40.7%)		0.571 (0.198, 1.653)
31–35 years	12 (46.2%)	14 (53.8%)		0.588 (0.260, 1.328)
Marital status				
Not married	165 (61.3%)	104 (38.7%)	< 0.001	1
Married	2 (11.1%)	16 (88.9%)		12.692 (2.860, 56.335)
Having children				
None	166 (58.8%)	118 (41.5%)	0.380	1
One or more	1 (33.3%)	2 (66.7%)		2.814 (0.252, 31.390)
Time since graduation				
More than 3 years	47 (87.0%)	7 (13.0%)	< 0.001	1
1–3 years	120 (51.5%)	113 (48.5%)		6.323 (2.744, 14.566)
Main income (baht)				
10 001–30 000	32 (65.3%)	17 (34.7%)	< 0.001	1
30 001–50 000	111 (86.7%)	17 (13.3%)		0.282 (0.129, 0.616)
Over 50 000	24 (27.0%)	65 (73.0%)		4.939 (2.323, 10.501)
Other income (baht)				
None	55 (44.0%)	70 (56.0%)	< 0.001	1
Less than 10 000	16 (64.0%)	9 (36.0%)		0.442 (0.182, 1.076)
10 000–30 000	42 (79.2%)	11 (20.8%)		0.211 (0.099, 0.448)
30 001–50 000	33 (86.8%)	5 (13.2%)		0.119 (0.044, 0.325)
Over 50 000	21 (84.0%)	4 (16.7%)		0.157 (0.051, 0.487)
OR = odds ratio

**Table 4:** Multivariable logistic regression model

Explanatory variables	*β*	SE	Adjusted OR	95% CI	*p* value
Sex					
Female	1.223	0.445	3.397	(1.421, 8.122)	0.006
Marital status					
Married	3.616	1.132	37.199	(4.048, 341.874)	0.001
Time since graduation					
1–3 years	2.805	0.669	16.522	(4.448, 61.366)	< 0.001
Main income (baht)					
10 001–30 000			1		
30 001–50 000	− 1.622	0.480	0.197	(0.077, 0.506)	0.001
Over 50 000	1.639	0.499	5.148	(1.936, 13.691)	0.001
Other income					
None			1		
< 10 000	− 1.28	0.603	0.278	(0.085, 0.907)	0.034
10 000–30 000	− 1.271	0.498	0.280	(0.106, 0.745)	0.011
30 001–50 000	− 1.763	0.667	0.172	(0.046, 0.634)	0.008
Over 50 000	− 1.881	0.77	0.152	(0.034, 0.690)	0.015

The principal stated factors influencing dentists’ retention or resignation from Thai government service were ranked by their cumulative point score. “Security in the profession” was the most important reason for retention, followed by “high chance to pursue specialty training in the future” and “close proximity to their hometown,” whereas “workplace far away from hometown” was the most important reason for resignation (Table 5). Reasons influencing retention and resignation from Thai government service for dentists recruited from the admission tracks are shown in Tables 6 and 7, which revealed that “security in the profession” was the most important reason for retention in the CURA, CUAS, and DACD, while “workplace far away from hometown” was the most important reason for resignation in the DCTMD and CUAS.

**Table 5:** Top reasons influencing the retention or resignation of recent dental graduates from CUSD from Thai government service

Rating	Reasons influencing retention in Thai government service	Reasons influencing resignation from Thai government service
1	Security in the profession	Workplace far away from hometown
2	High chance to pursue specialty training in the future	Getting specialty training
3	Close proximity to hometown	Unsatisfactory relationship with superiors and colleagues

**Table 6:** Reasons of recent dental graduates from CUSD in each admission track influencing retention in Thai government service

Admission tract	First rating	Second rating	Third rating
DCTMD	High chance to pursue specialty training in the future	Security in the profession	Satisfactory relationship with superiors and colleagues
CPIPRD	Close proximity to hometown	High chance to pursue specialty training in the future	Security in the profession
CURA	Security in the profession	Close proximity to hometown	Income satisfaction
CUAS	Security in the profession	High chance to pursue specialty training in the future	Satisfaction with welfare
DACD	Security in the profession	High chance to pursue specialty training in the future	Satisfaction with welfare
ASSP	**–**	**–**	**–**

**Table 7:** Reasons of recent dental graduates from CUSD in each admission track influencing resignation from Thai government service

Admission track	First rating	Second rating	Third rating
DCTMD	Workplace far away from hometown	Getting specialty training	Unsatisfactory relationship with superiors and colleagues
CPIPRD	Lack of freedom at work	Workplace far away from hometown	Getting specialty training
CURA	Unsatisfactory relationship with superiors and colleagues	Income dissatisfaction	Having their own private dental clinic
CUAS	Workplace far away from hometown	Getting specialty training	Unsatisfactory relationship with superiors and colleagues
DACD	Getting specialty training	Workplace far away from hometown	Unsatisfactory relationship with superiors and colleagues
ASSP	**–**	**–**	**–**

Focusing on the hometown of the high school students who entered and graduated from CUDS, three admission tracks (CPIPRD, CURA, and ASSP) recruited students from rural areas and CURA graduated dentists were sent back to work near their hometown after graduation. Therefore, the admission tracks were divided into two grouping tracks, following the regional backgrounds of most of the dental students in each track. The CPIPRD and CURA were combined into the first group (ASSP was excluded because there were no respondents from this track in this study), and the other tracks formed the second group. The factors that significantly influenced retention or resignation from the Thai government service in the CPIPRD and CURA tracks were “close proximity to hometown” and “satisfaction with welfare.” In contrast, “workplace far away from hometown” and “getting specialty training” were the two factors significantly associated with resignation from Thai government service (Additional file 1).

## Discussion

In this study, the retention rate of dentists within 7 years post-graduation was 58.2%, which was a low percentage; however, it was similar to the findings of Suphawirotloet et al. [12], who reported that dentists who worked less than 3 years tended to resign from Thai government service. Our findings were similar to the 10-year retention rate (53.3%) previously reported in a cross-sectional study [7] and suggests that the MoPH policy to increase the number and distribution of dentists in Thai government service is ineffective. This low percentage may reflect the low amount of compensation, approximately US$ 12 500, required to pay to resign from the government section before 3 years after graduation. Moreover, most of the students in the CUDS came from an urban area and tended to resign from rural work placements.

The general characteristics of the dentists who resigned from Thai government service which were significantly different from those of the retained dentists were being female, married, graduated less than 3 years, and income. Income and marriage were previously found to be influential factors in retaining dentists in Thai government service in Northerneast Thailand [13, 14]. The three most selected reasons for retention in Thai government service were security in the profession, high chance to pursue specialty training in the future, and close proximity to hometown. In contrast, the most selected reasons for resignation were workplace far away from hometown, getting specialty training, and an unsatisfactory relationship with superiors and colleagues. Thus, the relationship between the hometown and workplace was a potentially strong influence in the decision to stay or to resign. This result correlated with a previous study [15], which investigated the attitudes of Thai medical, dental, and pharmacy graduates towards working in rural areas and found that close proximity to their hometown was the most often selected factor in workplace choice. Moreover, dentists whose workplace was far away from their hometown had a greater tendency to resign from Thai government service [12]. In addition, our study also found that dentists who graduated from the CPIPRD and CURA admission tracks had a high proportion of retention in Thai government service compared with the other tracks. This result correlated with a previous study [10] that investigated the rural retention of doctors graduating from the rural medical education project to increase rural doctors in Thailand and found that the CPIRD track had a higher retention rate compared with the normal track.

The CPIPRD and CURA admission tracks could potentially have a high success rate in recruiting dentists to regional and rural areas, especially CURA, if placed nearer to their hometown and as a result of contract bonding, where they have to work in the MoPH hospital in their regional areas after graduation for 3 years or pay the release fee, as mentioned earlier. Hence, the effective policies of the Praboromarajchanok Institute under the MoPH to increase the retention level of dentists in Thai government service and solve the inequity distribution could be to recruit dental students from regional and rural backgrounds using a special contract bonding admission tract through the CPIPRD and CURA, with higher payback fees from leaving before 3 or perhaps 5 years after graduation. Moreover, the optional policy, which gives priority to graduated dentists to select their workplace in the same province or nearby their hometown, may be an effective strategy to increase the retention of dentists in rural areas.

Security in the profession was a key deciding factor for working in the public sector, which corresponds with previous studies that found security in the profession and income were significant factors influencing the decision concerning staying in Thai government service [12–14]. New medical graduates who work in rural areas automatically gain additional income on top of their regular salary, offsetting their opportunity loss for choosing to work in a rural area. Moreover, the Thai government’s civil servant medical benefits included in this employee’s health care system also covers their family. The MoPH should develop special policies to attract dentists to work in rural areas. A bundle of supporting mechanisms, such as promoting supportive work environments, enhancing financial incentives, and civil servant medical benefits for dentists, should be set up in parallel. This strategy has been very successful in retaining some dentists to work in rural areas.

Interestingly, getting specialty training influenced both the retention and resignation decisions and highlights a difference in perceived specialty training in Thai government service versus private practice between dentists and/or locations. A doctor of dental surgery degree provides training in basic dental care; however, dentists who work in community hospitals commonly face complicated cases that exceed their ability. These cases are typically referred to as the regional hospital that has specialists to provide appropriate treatment, leading to a backlog of patients and delayed treatment. Therefore, by receiving additional training during their working years, they might feel more confident in performing clinical work in a rural setting and feel confident in handling all types of dental patients and to seek professional peer support in the future.

Policy-makers could benefit from the results of this study. A special admission track program, CPIPRD and CURA, may help increase the number of dentists serving rural communities. Moreover, dental schools should support a curriculum that has more emphasis on dental public health, including community dentistry. This is necessary to prepare students for the compulsory work they must undertake in a public hospital after graduation.

This study has some limitations. Graduated dentists enrolled in the survey did not represent all the students graduating in 2005–2011, only approximately 30% of the total and none from the special APSS admission tract. However, the number of respondents was greater than the calculated minimum required sample size. One important consideration is the limitations of the design and data collection. Because this study is a cross-sectional survey using questionnaires, the results shown here are a static picture that cannot describe changes or trends in factors affecting the retention or resignation of dentists, and it may not reflect the real factors. To obtain a better understanding, a qualitative study, such as in-depth interviews, from other Thai universities and dental schools, is recommended for further study. Furthermore, the present study collected data from CU graduates between 2010 and 2016 because this period had the greatest number of admission tracks. However, most respondents were in the 3-year compulsory public service (81.2%) group. Thus, there were limitations about analyzing the differences in retention between the rural track and normal track within the first 3 years of work in public service and after 3 years of public work because of the low number of respondents after 3 years of public work. Although each dental school in Thailand has its own admission tracks, the major admission tracks for all public dental schools in Thailand during 2005–2011 were similar, including the CUAS, DCTMD, and CPIPRD. Other admission tracks depended on the policy of each university; however, these comprised a low number of dental students. Therefore, the results of this study may be generalized to other dental schools in Thailand.

## Conclusion

Dentists in the rural track had a significantly higher retention rate compared with the normal track. The most important factor influencing both retention and resignation was the workplace location relative to their hometown, where a workplace near their hometown reinforced the retention rate of rural dentists. Therefore, the MoPH should support a rural admission track to solve the inequity in dentist distribution, and dental schools should provide a curriculum that has more emphasis on dental public health, including community dentistry.

## Supplementary information

**Additional file 1: Table S1.** Factors influencing retention in the Thai government services in comparison between two admission tract groups. **Table S2.** Factors influencing from the Thai government services in comparison between two.

**Additional file 2.** Research questionnaire.

## Abbreviations

ASSP: Admission for Students in the Southern Border Provinces
COMES: Consortium of Thai Medical Schools
CPIPRD: Collaborative Project to Increase Production of Rural Dentists program
CU: Chulalongkorn University
CUAS: Central University Admissions System
CUDS: Chulalongkorn University Dental School
CURA: CU Rural Admission
DACD: Direct Admission for CUDS
DCTMD: Direct admission for the Consortium of Thai Medical and Dental School
MoPH: Ministry of Public Health of Thailand
OHEC: Office of Higher Education Commission
