# Supplementary material for: Dental school tracks related to the retention of dentists in Thai government service: a cross-sectional survey
Source: Hum Resour Health. 2020 Jan 28;18:5. doi: 10.1186/s12960-020-0444-7 (PMC6988324; doi:10.1186/s12960-020-0444-7)
Supplement: Supplementary file 1 — Additional file 1: Table S1. Factors influencing retention in the Thai government services in comparison between two admission tract groups. Table S2. Factors influencing from the Thai government services in comparison between two. [file 12960_2020_444_MOESM1_ESM.docx]

| **Table 7** Factors influencing retention in the Thai government services in comparison between two admission tract groups   \| Factor \| First track group^a^ \| Second track group^b^ \| P-value \| \| --- \| --- \| --- \| --- \| \| *Gender* \|  \|  \|  \| \| Male \| 16 (27.1%) \| 43 (72.9%) \| 0.665 \| \| Female \| 26 (24.1%) \| 82 (75.9%) \|  \| \| *Marital status* \|  \|  \|  \| \| Not married \| 42 (25.5%) \| 123 (74.4%) \| 0.41 \| \| Married \| 0 \| 2 (100.0%) \|  \| \| *Main income* \|  \|  \|  \| \| 10,001 – 30,000 \| 4 (12.5%) \| 28 (87.5%) \| 0.151 \| \| 30,001 – 50,000 \| 30 (27.0%) \| 81 (73.0%) \|  \| \| Over 50,000 \| 8 (33.3%) \| 16 (66.7%) \|  \| \| *Other income* \|  \|  \|  \| \| None \| 12 (21.8%) \| 43 (78.2%) \| 0.352 \| \| 10,000 – 30,000 \| 12 (20.7%) \| 46 (79.3%) \|  \| \| 30,001 – 50,000 \| 12 (36.4%) \| 21 (63.6%) \|  \| \| Over 50,000 \| 6 (28.6%) \| 15 (71.4%) \|  \| \| *Close proximity to hometown* \| \| \|  \| \| First rating \| 21 (53.8%) \| 18 (46.2%) \| <0.001* \| \| Second rating \| 7 (41.2%) \| 10 (58.8%) \|  \| \| Third rating \| 2 (10.5%) \| 17 (89.5%) \|  \| \| *Satisfaction with income* \| \| \|  \| \| First rating \| 3 (50.0%) \| 3 (50.0%) \| 0.084 \| \| Second rating \| 2 (18.2%) \| 9 (81.8%) \|  \| \| Third rating \| 3 (50.0%) \| 3 (50.0%) \|  \| \| *Security in the profession* \| \| \|  \| \| First rating \| 8 (24.2%) \| 25 (75.8%) \| 0.753 \| \| Second rating \| 10 (25.6%) \| 29 (74.4%) \|  \| \| Third rating \| 6 (%) \| 27 (%) \|  \| \| *Satisfactory relationship with leaderships and colleagues* \| \| \|  \| \| First rating \| 1 (5.3%) \| 18 (94.7%) \| 0.115 \| \| Second rating \| 6 (27.3%) \| 16 (72.3%) \|  \| \| Third rating \| 7 (35.0%) \| 13 (65.0%) \|  \| \| *Satisfaction with welfare* \| \| \|  \| \| First rating \| 2 (9.5%) \| 19 (90.5%) \| 0.044* \| \| Second rating \| 3 (12.5%) \| 21 (87.5%) \|  \| \| Third rating \| 9 (40.9%) \| 13 (59.1%) \|  \| \| *Advancement in the profession* \| \| \|  \| \| First rating \| 0 \| 1 (100.0%) \| 0.066 \| \| Second rating \| 5 (55.5%) \| 4 (44.5%) \|  \| \| Third rating \| 2 (20.0%) \| 8 (80.0%) \|  \| \| *Independence at work* \|  \|  \|  \| \| First rating \| 1 (12.5%) \| 7 (87.5%) \| 0.357 \| \| Second rating \| 3 (15.8%) \| 16 (84.2%) \|  \| \| Third rating \| 1 (5.9%) \| 16 (94.1%) \|  \| \| *High chance to pursue specialty training in the future* \| \| \|  \| \| First rating \| 6 (17.1%) \| 29 (82.9%) \| 0.282 \| \| Second rating \| 5 (25.0%) \| 15 (75.0%) \|  \| \| Third rating \| 11 (32.4%) \| 23 (67.6%) \|  \| \| ^a^First group, rural admissions, was comprised of CPIPRD and CURA (no participants from ASSP) \| \| \| \| \| ^b^Second group, urban admissions, was comprised of DACD, COMES and DCTMD.  **Table 8** Factors influencing from the Thai government services in comparison between two admission tract groups**.**   \| Factor \| First track group^a^ \| Second track group^b^ \| P-value \| \| --- \| --- \| --- \| --- \| \| *Gender* \|  \|  \|  \| \| Male \| 5 (29.4%) \| 12 (70.6%) \| 0.522 \| \| Female \| 23 (22.3%) \| 80 (77.7%) \|  \| \| *Marital status* \|  \|  \|  \| \| Not married \| 13 (12.5%) \| 91 (87.5%) \| 0.182 \| \| Married \| 4 (25.0%) \| 12 (75.0%) \|  \| \| *Number of children* \|  \|  \|  \| \| None \| 4 (28.6%) \| 10 (71.4%) \| 0.383 \| \| One or more \| 0 \| 2 (100.0%) \|  \| \| *Main income* \|  \|  \|  \| \| 10,001 – 30,000 \| 2 (11.8%) \| 15 (88.2%) \| 0.862 \| \| 30,001 – 50,000 \| 3 (17.6%) \| 14 (82.4%) \|  \| \| Over 50,000 \| 11 (16.9%) \| 54 (83.1%) \|  \| \| *Other income* \|  \|  \|  \| \| None \| 12 (17.1%) \| 58 (82.9%) \| 0.572 \| \| 10,000 – 30,000 \| 4 (20.0%) \| 16 (80.0%) \|  \| \| 30,001 – 50,000 \| 0 \| 5 (100.0%) \|  \| \| Over 50,000 \| 0 \| 4 (100.0%) \|  \| \| *Other occupation* \|  \|  \|  \| \| First rating \| 0 \| 1 (100.0%) \| 0.771 \| \| Second rating \| 0 \| 0 \|  \| \| Third rating \| 0 \| 1 (100.0%) \|  \| \| *Workplace far away from hometown* \| \|  \|  \| \| First rating \| 2 (5.7%) \| 33 (94.3%) \| 0.026* \| \| Second rating \| 2 (8.0%) \| 23 (92.0%) \|  \| \| Third rating \| 4 (20.0%) \| 16 (80.0%) \|  \| \| *Dissatisfaction with income* \|  \|  \|  \| \| First rating \| 1 (16.7%) \| 5 (83.3%) \| 0.294 \| \| Second rating \| 5 (38.5%) \| 8 (61.5%) \|  \| \| Third rating \| 1 (8.3%) \| 11 (91.7%) \|  \| \| *Unsatisfactory relationship with leaderships and colleagues* \|  \|  \|  \| \| First rating \| 4 (17.4%) \| 19 (82.6%) \| 0.211 \| \| Second rating \| 1 (7.1%) \| 13 (92.9%) \|  \| \| Third rating \| 0 \| 9 (100.0%) \|  \| \| *Lack of advancement opportunities* \|  \|  \|  \| \| First rating \| 2 (66.7%) \| 1 (33.3%) \| 0.052 \| \| Second rating \| 0 \| 11 (100.0%) \|  \| \| Third rating \| 1 (9.1%) \| 10 (90.9%) \|  \| \| *Lack of freedom at works* \|  \|  \|  \| \| First rating \| 2 (28.6%) \| 5 (71.4%) \| 0.396 \| \| Second rating \| 5 (31.2%) \| 11 (68.8%) \|  \| \| Third rating \| 0 \| 6 (100.0%) \|  \| \| *Getting a specialty training* \|  \|  \|  \| \| First rating \| 3 (8.8%) \| 31 (91.2%) \| 0.047* \| \| Second rating \| 1 (5.9%) \| 16 (94.1%) \|  \| \| Third rating \| 1 (8.3%) \| 11 (91.7%) \|  \| \| *Make their own private dental clinic* \|  \|  \|  \| \| First rating \| 0 \| 1 (100.0%) \| 0.845 \| \| Second rating \| 0 \| 1 (100.0%) \|  \| \| Third rating \| 1 (33.3%) \| 2 (66.7%) \|  \| \| *Hard workload* \|  \|  \|  \| \| First rating \| 1 (100.0%) \| 0 \| 0.114 \| \| Second rating \| 0 \| 4 (100.0%) \|  \| \| Third rating \| 0 \| 4 (100.0%) \|  \| \| *Limited facilities in rural areas* \|  \|  \|  \| \| First rating \| 0 \| 1 (100.0%) \| 0.421 \| \| Second rating \| 0 \| 5 (100.0%) \|  \| \| Third rating \| 1 (8.3%) \| 11 (91.7%) \|  \| \| *Health problems* \|  \|  \|  \| \| First rating \| 0 \| 1 (100.0%) \| 0.097 \| \| Second rating \| 1 (100.0%) \| 0 \|  \| \| Third rating \| 2 (50.0%) \| 2 (50.0%) \|  \| \| *Take care of the parents/children/married* \| \|  \|  \| \| First rating \| 1 (33.3%) \| 2 (66.7%) \| 0.599 \| \| Second rating \| 0 \| 6 (100.0%) \|  \| \| Third rating \| 3 (20.0%) \| 12 (80.0%) \|  \| \| ^a^First group, rural admissions, was comprised of CPIPRD and CURA (no participants from ASSP) \| \| \| \| \| ^b^Second group, urban admissions, was comprised of DACD, COMES and DCTMD. \| \| \| \| \|  \|  \|  \|  \| \| \| \| \| |
| --- | --- | --- | --- | --- | --- | --- | --- | --- | --- | --- | --- | --- | --- | --- | --- | --- | --- | --- | --- | --- | --- | --- | --- | --- | --- | --- | --- | --- | --- | --- | --- | --- | --- | --- | --- | --- | --- | --- | --- | --- | --- | --- | --- | --- | --- | --- | --- | --- | --- | --- | --- | --- | --- | --- | --- | --- | --- | --- | --- | --- | --- | --- | --- | --- | --- | --- | --- | --- | --- | --- | --- | --- | --- | --- | --- | --- | --- | --- | --- | --- | --- | --- | --- | --- | --- | --- | --- | --- | --- | --- | --- | --- | --- | --- | --- | --- | --- | --- | --- | --- | --- | --- | --- | --- | --- | --- | --- | --- | --- | --- | --- | --- | --- | --- | --- | --- | --- | --- | --- | --- | --- | --- | --- | --- | --- | --- | --- | --- | --- | --- | --- | --- | --- | --- | --- | --- | --- | --- | --- | --- | --- | --- | --- | --- | --- | --- | --- | --- | --- | --- | --- | --- | --- | --- | --- | --- | --- | --- | --- | --- | --- | --- | --- | --- | --- | --- | --- | --- | --- | --- | --- | --- | --- | --- | --- | --- | --- | --- | --- | --- | --- | --- | --- | --- | --- | --- | --- | --- | --- | --- | --- | --- | --- | --- | --- | --- | --- | --- | --- | --- | --- | --- | --- | --- | --- | --- | --- | --- | --- | --- | --- | --- | --- | --- | --- | --- | --- | --- | --- | --- | --- | --- | --- | --- | --- | --- | --- | --- | --- | --- | --- | --- | --- | --- | --- | --- | --- | --- | --- | --- | --- | --- | --- | --- | --- | --- | --- | --- | --- | --- | --- | --- | --- | --- | --- | --- | --- | --- | --- | --- | --- | --- | --- | --- | --- | --- | --- | --- | --- | --- | --- | --- | --- | --- | --- | --- | --- | --- | --- | --- | --- | --- | --- | --- | --- | --- | --- | --- | --- | --- | --- | --- | --- | --- | --- | --- | --- | --- | --- | --- | --- | --- | --- | --- | --- | --- | --- | --- | --- | --- | --- | --- | --- | --- | --- | --- | --- | --- | --- | --- | --- | --- | --- | --- | --- | --- | --- | --- | --- | --- | --- | --- | --- | --- | --- | --- | --- | --- | --- | --- | --- | --- | --- | --- | --- | --- | --- | --- | --- | --- | --- | --- | --- | --- | --- | --- | --- | --- | --- | --- | --- | --- | --- | --- | --- | --- | --- | --- | --- | --- | --- | --- | --- | --- | --- | --- | --- | --- | --- | --- | --- | --- | --- | --- | --- | --- | --- | --- | --- | --- | --- | --- | --- | --- | --- | --- | --- | --- | --- | --- | --- | --- | --- | --- | --- | --- | --- | --- | --- | --- | --- | --- | --- | --- | --- | --- | --- | --- | --- | --- | --- | --- | --- | --- | --- | --- | --- | --- | --- | --- | --- | --- | --- | --- | --- | --- | --- | --- | --- | --- | --- | --- | --- | --- | --- | --- | --- | --- | --- | --- | --- | --- | --- | --- | --- | --- | --- | --- | --- | --- | --- | --- | --- | --- | --- | --- | --- | --- | --- | --- | --- | --- | --- | --- | --- | --- | --- | --- | --- | --- |
