# Supplementary material for: Dental school tracks related to the retention of dentists in Thai government service: a cross-sectional survey
Source: Hum Resour Health. 2020 Jan 28;18:5. doi: 10.1186/s12960-020-0444-7 (PMC6988324; doi:10.1186/s12960-020-0444-7)
Supplement: Supplementary file 2 — Additional file 2. Research questionnaire. [file 12960_2020_444_MOESM2_ESM.pdf]

Researh: Tracks of admission for Chulalongkorn dental school during 2005-2011 related to retention of dentists in Thai government services

This research is a part of the subject: Research methodology for academic year 2017
Faculty of dentistry, Chulalongkorn university

Research objectives
1. To determine percentage of dentists in Thai government services who admit in academic year 2005-2011.
2. To compare number of dentists who work and resigned in the Thai governments services in each tracks of the dental school admission.
3. To identify the association of tracks of admission during academic year 2005-2011 and retention of dentists in Thai government services.
4. To examine the factor associated with retention and resignation from the Thai governments services of dentist from each tracks of the dental school admission.

Anticipated Benefit Gain
This study aims to identify the association between the tracks of admission for Chulalongkorn dental school and retention of dentists in government Thai services, which benefit future dental manpower planning in the rural areas.

Participant Information Sheet (จธ. 2.4)
Link : https://drive.google.com/open?id=184ZON9oZOSs8oxVehahYzJWJwDsXd8vs
Withdrawal Form (จธ. 2.6)
Link : https://drive.google.com/open?id=1EFqeiava_e7_sd_crxyqYDzhTOvM16Uq

*Required

-

1. **Do you read the participant information sheet and agree to participate in the research ?** *

Mark only one oval.

( ) Yes
( ) No *Stop filling out this form.*

You already read the participant information sheet and agree to participate in the research.

Part I

2. **Age (year)** *

Mark only one oval.

- 20-25
- 26-30
- 31-35
- 36-40
- > 40

3. **Gender** *

Mark only one oval.

- Male
- Female

4. **Marital status** *

Mark only one oval.

- Not married (Not answer in the "Number of children")
- Marriage and living together
- Marriage and not living together
- Divorced

5. **Number of children**

Mark only one oval.

- None
- 1
- 2
- more than 2

6. **The highest education** *

Mark only one oval.

- Doctor of Dental Surgery
- Graduate Diploma in Clinical Sciences
- Master of Science
- Higher Graduate Diploma in Clinical Sciences
- Doctor of Philosophy
- Residency Training Program
- Other: ____________________

7. **Hometown** *
Mark only one oval.

- Bangkok Metropolitan Region
- Northern Thailand
- Central Thailand
- Northeastern Thailand
- Westhern Thailand
- Southern Thailand

8. **Administration academic year in DDS** *
Mark only one oval.

- 2005
- 2006
- 2007
- 2008
- 2009
- 2010
- 2011

9. **Tracks of admission** *
Mark only one oval.

- Direct Admission for Consortium of Thai Medical and Dental school
- The Collaborative Project to Increase Production of Rural Dentists Program
- Chulalongkorn Rural Admission
- Direct Admission for Chulalongkorn Dental school
- Central University Admission System
- Admission for Students in the Southern Border Provinces, under the desire of Ministry of Interior

10. **Graduated academic year** *
Mark only one oval.

- 2010
- 2011
- 2012
- 2013
- 2014
- 2015
- 2016
- 2017

Part II

11. **Career** *

Mark only one oval.

- Dentist
- Dental professor
- Others

12. **Do you have your own private dental clinic or hospital ?** *

Mark only one oval.

- Yes
- No

13. **Where is your the most workplace in a month ?** *

Mark only one oval.

- Public health services *Skip to question 14.*
- Ministry of University Affairs *Skip to question 21.*
- Private health services *Skip to question 32.*
- Private university *Skip to question 27.*
- Other: ______________ *Skip to question 42.*

Public health services

14. **Affiliation of work** *

Mark only one oval.

- Ministry of Public Health
- Ministry of Defence
- Ministry of Inferior
- Other: ______________

15. **Is your hometown and workplace as in the same provinces ?** *

Mark only one oval.

- Yes
- No

16. **Is your workplace's province has an airport ?** *

Mark only one oval.

- Yes
- No
- Nearby

17. **Total income from government services (Thai bath)** *

Mark only one oval.

- 10,001 - 20,000
- 20,001 - 30,000
- 30,001 - 40,000
- 40,001 - 50,000
- Over 50,000

18. **Other income (Thai bath)** *

Mark only one oval.

- None
- Under 10,000
- 10,001 - 20,000
- 20,001 - 30,000
- 30,001 - 40,000
- 40,001 - 50,000
- Over 50,000
- Other: ______________________

19. **Hospital size (Number of bed)** *

Mark only one oval.

- 30
- 60
- 90
- 120
- Over 120

20. **Number of dentists in the hospital that you work (including you)** *

Mark only one oval.

- 1
- 2
- 3
- 4
- 5
- 6
- 7
- 8
- 9
- 10
- Over 10

Skip to question 36.

Ministry of University Affairs

21. **Affiliation of work** *
Mark only one oval.

- Chulalongkorn university
- Mahidol university
- Thammasat university
- Srinakharinwirot university
- Khonkaen university
- Chiang Mai University
- Naresuan university
- Prince of Songkla University
- University of Phayao
- Mae Fah Luang University
- College of public health, Ministry of Public Health
- Suranaree University of Technology
- Burapha University

22. **Academic ranks** *
Mark only one oval.

- Lecturer
- Assistant professor
- Associate professor
- Professor
- General dental practitioner in university services

23. **Is you have a contract-bonding from Thai government service or university ?** *
Mark only one oval.

- Yes
- No

24. **Is your hometown and workplace as in the same provinces ?** *
Mark only one oval.

- Yes
- No

25. **Total income from university (Thai bath)** *
Mark only one oval.

- 10,001 - 20,000
- 20,001 - 30,000
- 30,001 - 40,000
- 40,001 - 50,000
- Over 50,000

26. **Other income (Thai bath)** *
Mark only one oval.

- None
- under 10,000
- 10,001 - 20,000
- 20,001 - 30,000
- 30,001 - 40,000
- 40,001 - 50,000
- Over 50,000

Skip to question 36.

Private University

27. **Affiliation of work**
Mark only one oval.

- Rangsit university
- Western university

28. **Academic ranks** *
Mark only one oval.

- Lecturer
- Assistant professor
- Associate professor
- Professor
- General dental practitioner in university services

29. **Is you have a contract-bonding from university ?** *
Mark only one oval.

- Yes
- No

30. **Total income from university (Thai bath)** *
Mark only one oval.

- 10,001 - 20,000
- 20,001 - 30,000
- 30,001 - 40,000
- 40,001 - 50,000
- Over 50,000

31. **Other income (Thai bath)** *
Mark only one oval.

- None
- Under 10,000
- 10,001 - 20,000
- 20,001 - 30,000
- 30,001 - 40,000
- 40,001 - 50,000
- Over 50,000

Skip to question 42.

Private health services

32. **Total income from private hospital (Thai bath)** *
Mark only one oval.

- 10,001 - 20,000
- 20,001 - 30,000
- 30,001 - 40,000
- 40,001 - 50,000
- Over 50,000

33. **Other income (Thai bath)** *
Mark only one oval.

- None
- Under 10,000
- 10,001 - 20,000
- 20,001 - 30,000
- 30,001 - 40,000
- 40,001 - 50,000
- Over 50,000

34. **Type of private services** *
Mark only one oval.

- Private hospital
- Private clinic

35. **Number of dentists in the hospital that you work (including you)** *
Mark only one oval.

- 1
- 2
- 3
- 4
- 5
- 6
- 7
- 8
- 9
- 10
- Over 10

Skip to question 42.

Part III

36. **What is your first workplace after graduated from DDS program** *
Mark only one oval.

- Public health services
- MInistry of University Affairs
- Private health services
- Private university
- Other: ____________________

37. **Duration of work in Thai government services (Year)** *

____

38. **Have you ever changed the workplace? (If you answer "ever", answer in the next question, please)**
Mark only one oval.

- ever
- never

39. **What is the reasons to change workplace?**

____

40. **The reasons influencing retention in the Thai government services (Please select 3 position, in order of 1 from top to bottom.)**

* If you have other reasons, you can answer in the next section.

Tick all that apply.

	The 1st rating	The 2nd rating	The 3rd rating
Close proximity to hometown	☐	☐	☐
income	☐	☐	☐
Security in the profession	☐	☐	☐
Advancement in the profession	☐	☐	☐
Satisfaction with relationship with leaderships and colleagues	☐	☐	☐
Satisfaction with welfare	☐	☐	☐
Freedom at works	☐	☐	☐
High chance to pursue specialty training in the future	☐	☐	☐

41. **Other reasons**

____

Part IV

42. **What is your first workplace after graduated from DDS program** *

Mark only one oval.

- ◯ Public health services
- ◯ MInistry of University Affairs
- ◯ Private health services
- ◯ Private university
- ◯ Other: ________________________

43. **Duration of work in Thai government services (Year)**

____

44. **The reasons influencing resignation from the Thai government services (Please select 3 position, ranked 1 for each reason.)**

Tick all that apply.

	The 1st rating	The 2nd rating	The 3rd rating
Workplace far away from hometown	☐	☐	☐
limited facilities in rural areas	☐	☐	☐
Dissatisfaction with income	☐	☐	☐
Dissatisfaction with relationship with leaderships and colleagues	☐	☐	☐
Lack of advancement opportunities	☐	☐	☐
Lack of freedom at works	☐	☐	☐
Hard workload	☐	☐	☐
Getting a specialty training	☐	☐	☐
Make their own private dental clinic	☐	☐	☐
Other occupation	☐	☐	☐
Health problems	☐	☐	☐
Take care of the parents/children/married	☐	☐	☐

45. **Other reasons**

____

Powered by
Google Forms
